# Supplementary material for: Research Progress on Elements of Wild Edible Mushrooms
Source: J Fungi (Basel). 2022 Sep 15;8(9):964. doi: 10.3390/jof8090964 (PMC9505289; doi:10.3390/jof8090964)
Supplement: Supplementary file 1 [file jof-08-00964-s001.zip › jof-1903387-supplementary.pdf]

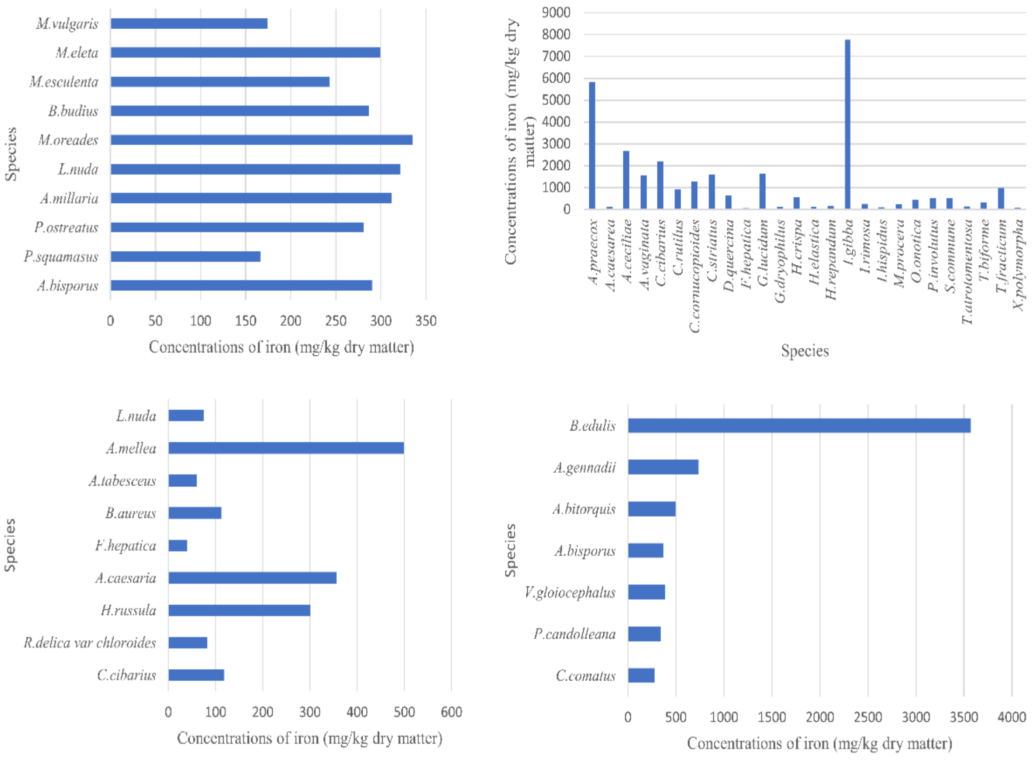

Figure S1. The comparative graph of iron content.

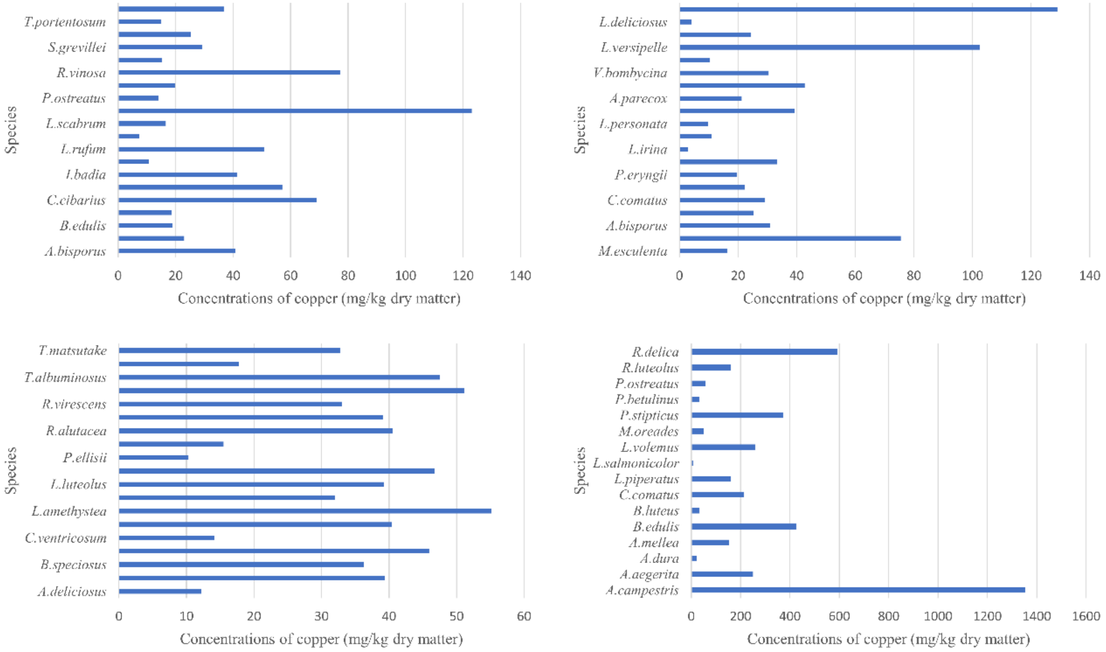

Figure S2. The comparative graph of copper content.

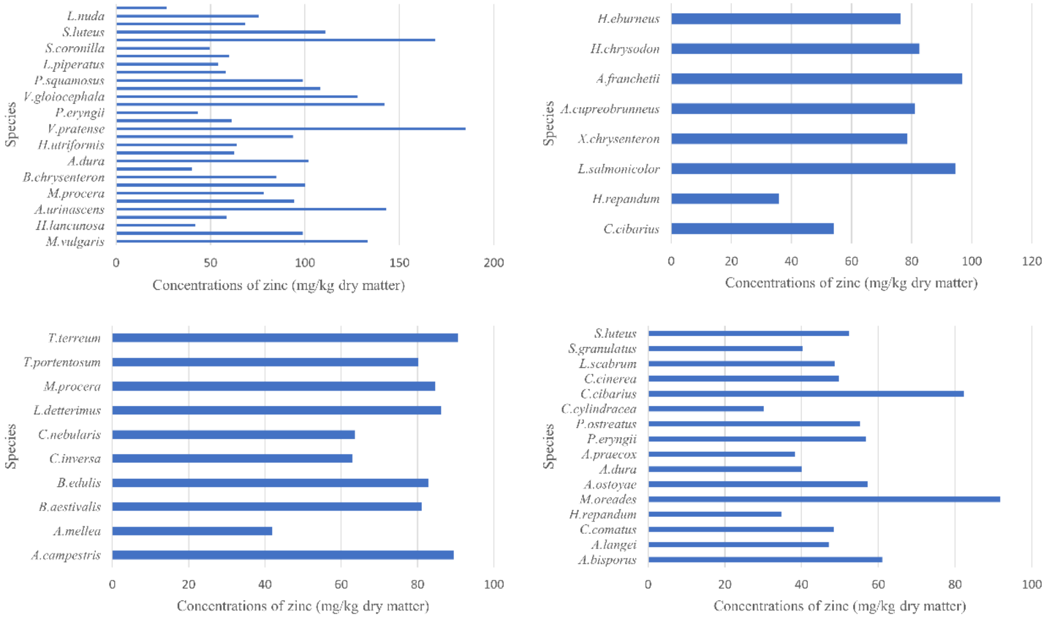

Figure S3. The comparative graph of zinc content.

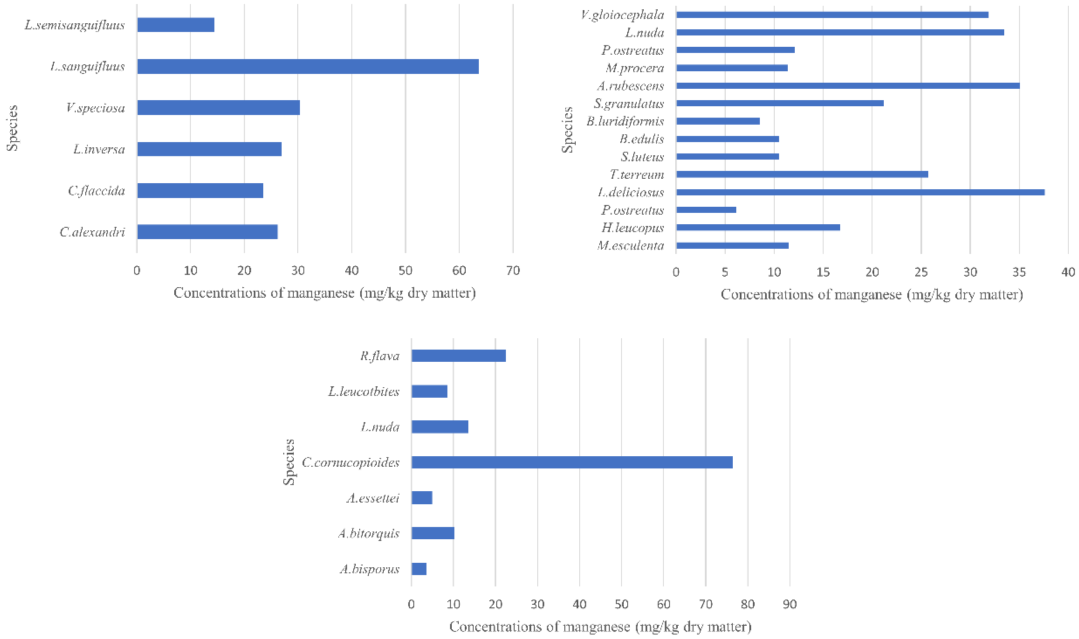

Figure S4. The comparative graph of manganese content.

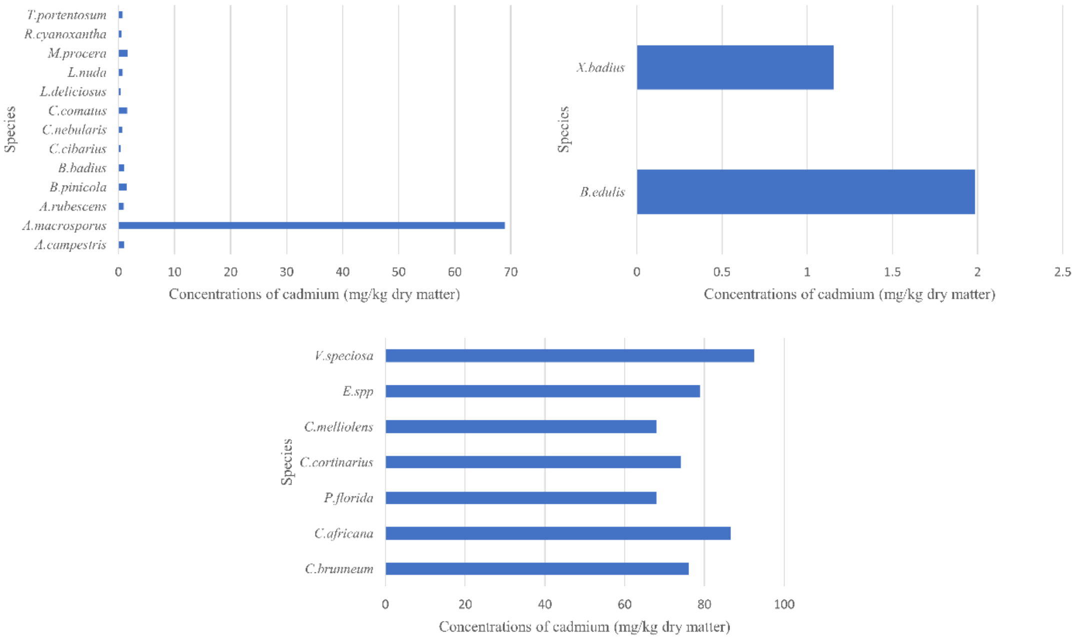

Figure S5. The comparative graph of cadmium content.

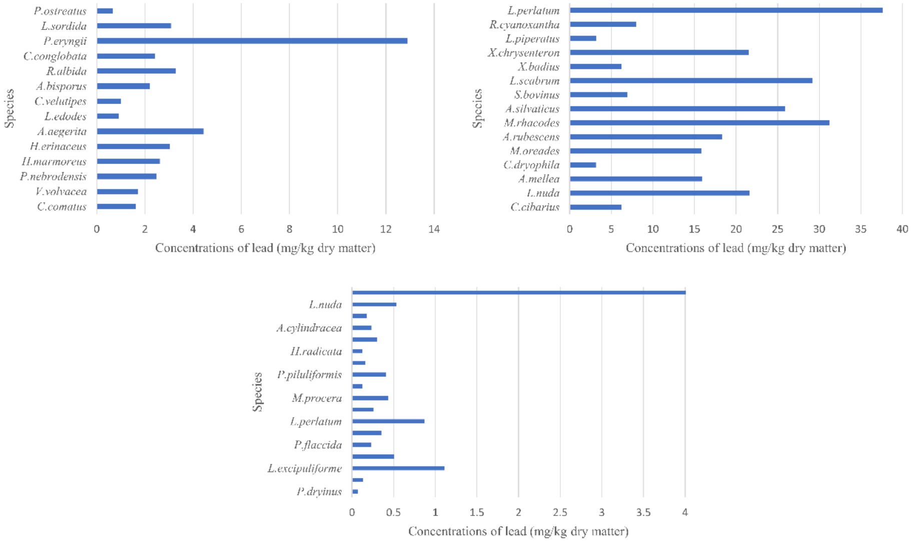

Figure S6. The comparative graph of lead content.

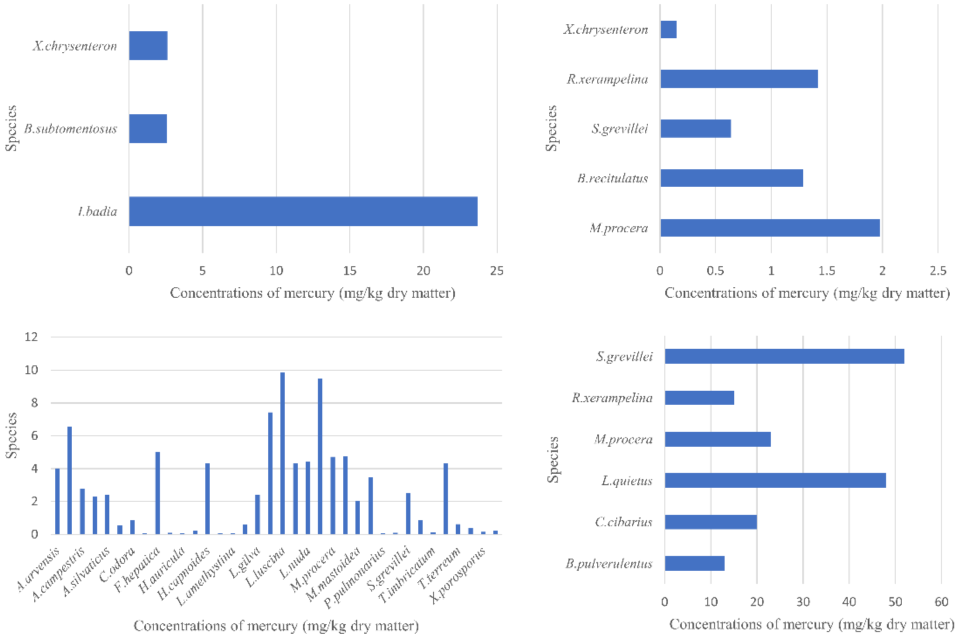

Figure S7. The comparative graph of mercury content.

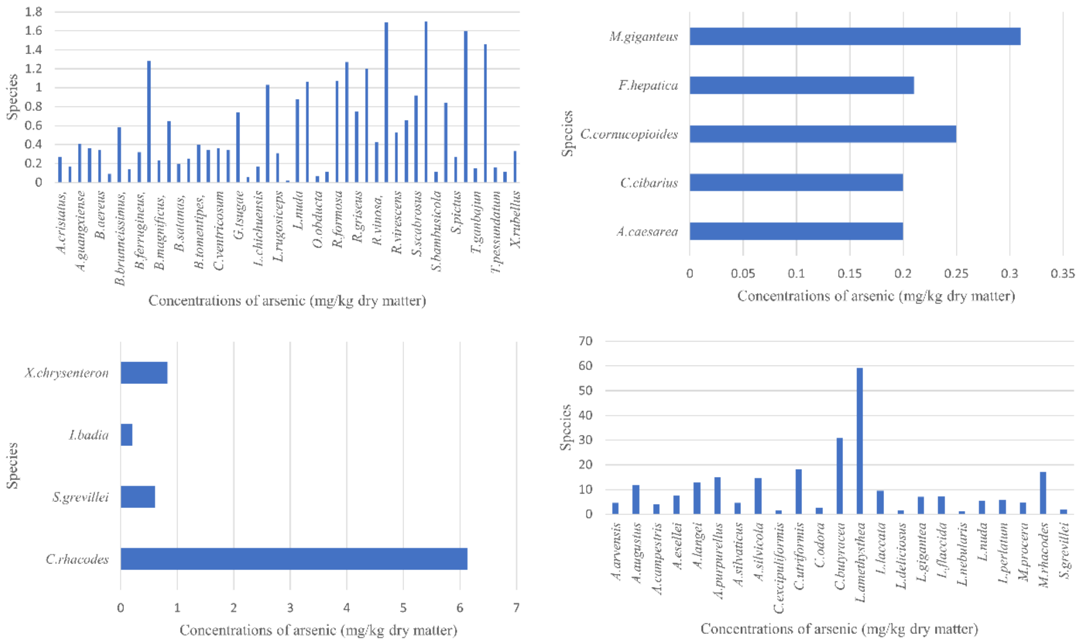

Figure S8. The comparative graph of arsenic content.
